# Psychological distress and incident cardiovascular disease independent of life’s essential 8: a prospective cohort study

**DOI:** 10.1080/07853890.2026.2665515

**Published:** 2026-05-04

**Authors:** Nami Lee, Kyoung Hwa Ha, Hyun Young Lee, Sang Joon Son, Seung Jin Han

**Affiliations:** a Department of Endocrinology and Metabolism, Ajou University School of Medicine, Suwon, Republic of Korea; b Clinical Trial Center, Ajou University Hospital, Suwon, Republic of Korea; c Department of Psychiatry, Ajou University School of Medicine, Suwon, Republic of Korea

**Keywords:** Cardiovascular health metrics, psychological distress, cardiovascular diseases

## Abstract

**Background:**

Although psychological stress has emerged as an important determinant of cardiovascular disease (CVD) risk, it remains excluded from the recently updated cardiovascular health (CVH) metrics, known as Life’s Essential 8 (LE8). This study aimed to examine the association between psychological distress and the incidence of CVD, independent of Life’s Essential 8 metrics, in a large Korean adult population.

**Methods:**

This study included 6,410 participants from the Korean Genome and Epidemiology Study Ansan–Ansung cohort, who had no history of CVD and had complete baseline data on psychological distress and Life’s Essential 8 cardiovascular health (LE8 CVH) metrics. Psychological distress was assessed using the Psychosocial Wellbeing Index Short Form (PWI-SF). CVD events were identified based on participants’ self-reports of physician-diagnosed conditions: myocardial infarction, stroke, coronary artery disease, and congestive heart failure. Cox proportional hazards models were used to examine the association between PWI-SF scores and incident CVD, adjusting for age, sex, residential area, educational attainment, household income, and LE8 CVH metrics.

**Results:**

During a median follow-up of 13.8 years, 500 new cases of CVD were identified. Higher PWI-SF scores were independently associated with an increased risk of CVD after adjusting for LE8 CVH metrics and other potential confounders (hazard ratio: 1.321; 95% confidence interval: 1.067–1.636; *p* = 0.011).

**Conclusion:**

These findings suggest that higher levels of psychological distress are independently associated with an increased risk of CVD, even after accounting for established LE8 CVH metrics. Incorporating psychological distress into future CVH assessments may enhance risk stratification and prevention strategies.

## GRAPHICAL ABSTRACT

**Figure:**
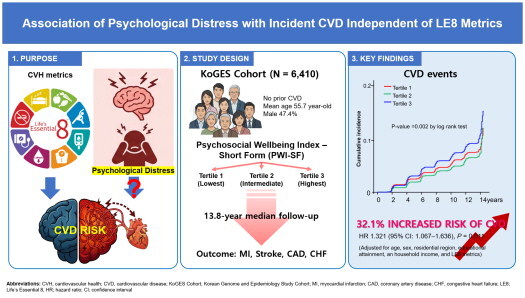

## 1. Introduction

Cardiovascular disease (CVD) remains the leading cause of morbidity and mortality worldwide. According to the World Health Organization (2022), CVDs were responsible for an estimated 17.9 million deaths, accounting for approximately 32% of global fatalities [1]. Despite significant advancements in both prevention and treatment, CVD continues to represent a major public health concern. Consequently, recent cardiovascular epidemiology research has increasingly focused on identifying emerging and modifiable CVD risk factors within the general population.

Psychological stress is a broad construct describing experiences in which environmental demands exceed an individual’s perceived psychological and physiological capacity to cope effectively [2]. In stress research, it is important to distinguish between exposure to stressors and the responses elicited by those stressors. Stressors refer to external events or conditions that may disrupt normal functioning, whereas stress responses encompass psychological, cognitive, behavioral, and physiological reactions that occur in response to such exposures [3,4]. Stressors may occur across different time scales, including acute stressors, daily hassles, major life events, and chronic stressors. Among the various responses to stressor exposure, psychological distress represents a psychological state characterized by a perceived inability to cope effectively, changes in emotional status, subjective discomfort, and the expression of distress [5]. In the present study, we focus on psychological distress, which reflects a chronic subjective state rather than acute stress responses, as assessed using the Psychosocial Well-being Index Short Form (PWI-SF).

A growing body of evidence has linked psychological stress to an increased risk of CVD, including earlier disease onset, worse clinical outcomes, and elevated mortality risk [6,7]. Large international studies such as the INTERHEART and INTERSTROKE studies primarily examined exposure to psychosocial stressors, including stress at work or home, financial stress, and major life events, and demonstrated significant associations with acute myocardial infarction and stroke across diverse populations [8,9]. In addition to studies focusing on stressor exposure, prospective cohort studies have reported that psychological distress, assessed using brief mental health screening instruments, is associated with an increased risk of CVD and CVD-related mortality [10–12].

In an effort to promote cardiovascular health (CVH) and prevent CVD, the American Heart Association (AHA) introduced the concept of ideal CVH in 2010, known as ‘Life’s Simple 7,’ which included seven health behaviors and health factors: smoking status, physical activity, diet, body weight, blood pressure, blood glucose, and cholesterol levels [13]. In 2022, the AHA updated these metrics as ‘Life’s Essential 8 (LE8)’ by adding sleep health as a new component and introducing a refined 0–100 scoring system to allow for more precise and continuous assessment of CVH status [14]. Although psychological health and well-being have been increasingly recognized as important contributors to CVH, they were not included as formal components of the LE8 CVH metrics. The AHA scientific statement noted that psychological health is multidimensional and interacts with multiple aspects of CVH, and therefore was considered a foundational factor underlying CVH rather than a distinct metric itself. Following the release of the updated LE8 guidelines in 2022, higher LE8 scores have been associated with a lower risk of CVD [15].

However, it remains unclear whether psychological distress contributes to CVD risk independently of the established LE8 CVH metrics. Clarifying this relationship may improve cardiovascular risk stratification and highlight the potential value of incorporating psychological distress into cardiovascular prevention strategies. We hypothesized that higher levels of psychological distress are associated with an increased risk of CVD independent of LE8 CVH metrics. Therefore, we examined whether psychological distress, assessed using the PWI-SF, is associated with incident CVD independent of LE8 CVH metrics in a Korean community-based prospective cohort with a 14-year follow-up period.

## 2. Material and methods

### 2.1. Study population

We used the data from the Korean Genome and Epidemiology Study (KoGES) Ansan-Ansung, an ongoing, community-based prospective cohort study, conducted by the Korea National Institute of Health [16]. The Ansan–Ansung cohort was established between 2001 and 2002 to investigate the interplay between genetic predispositions, environmental exposures, and lifestyle determinants in the development of chronic diseases among Koreans. This study is a longitudinal analysis of the KoGES Ansan–Ansung cohort, in which the second follow-up examination (2005–2006) served as the baseline, and participants were followed up through the ninth examination (2019–2020) to assess outcomes. The cohort enrolled 10,030 individuals aged 40–69 years from two demographically distinct regions: Ansan (urban) and Ansung (rural), ensuring a representative sample of the middle-aged Korean population. Participants provided a wide range of information, including age, sex, anthropometric measurements, and lifestyle factors, through self-reported questionnaires, clinical examinations, and medical records. Follow-up examinations were conducted biennially between 2003 and 2020. The study protocol was approved by the Institutional Review Board of the Korea Centers for Disease Control and Prevention. All participants provided written informed consent, and all research procedures were performed in accordance with the relevant guidelines and regulations. The study was conducted in accordance with the principles of the Declaration of Helsinki and was approved by the Institutional Review Board of Ajou University (AJOUIRB-EX-2024-307).

**Figure 1.**
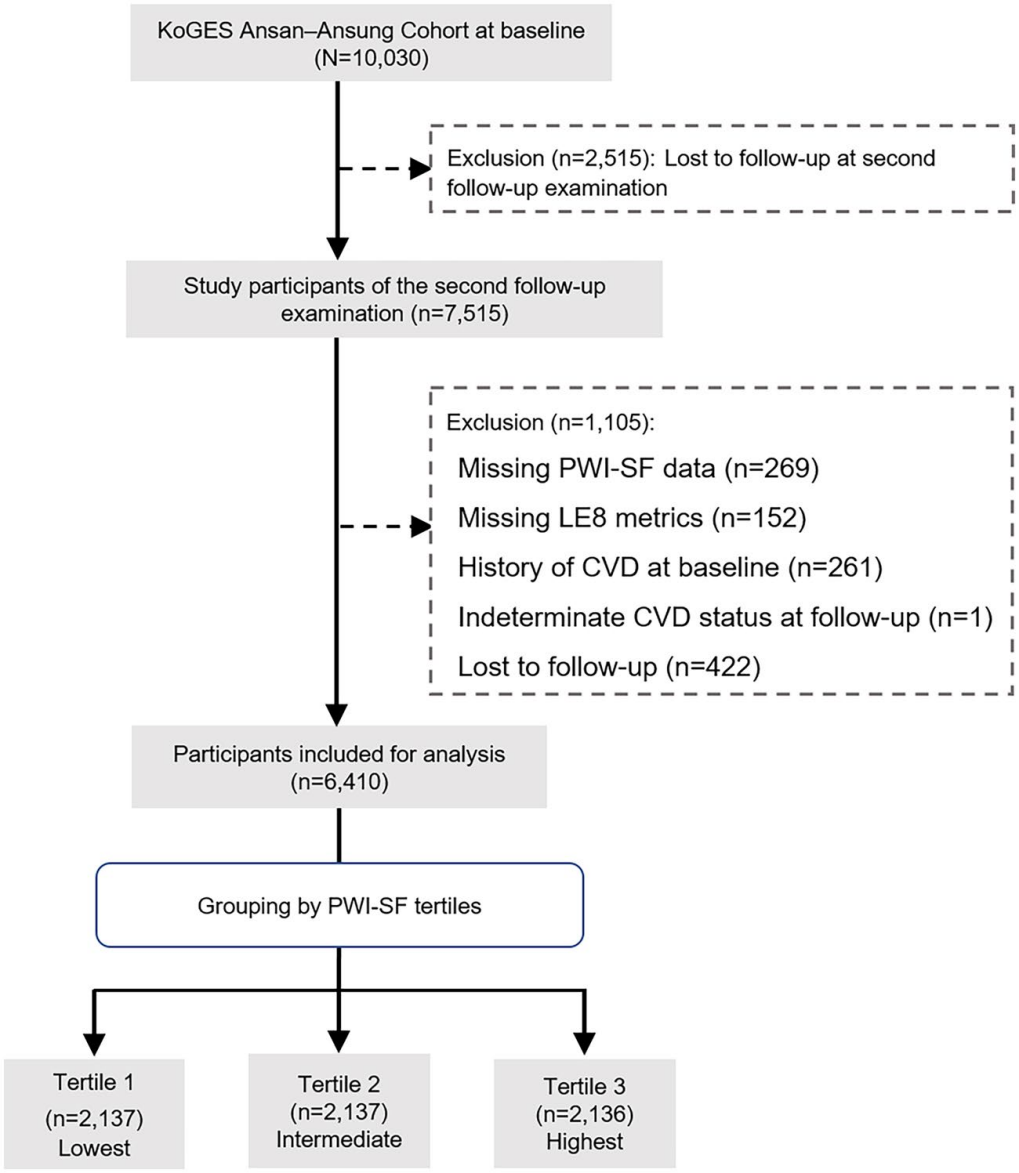
Flowchart of study participant selection and exclusion. KoGES, Korean Genome and Epidemiology Study; PWI-SF, Psychosocial Well-being Index Short Form; LE8, Life’s Essential 8; CVD, cardiovascular disease.

This study adhered to the Strengthening the Reporting of Observational Studies in Epidemiology guidelines. Of the 10,030 participants initially enrolled in the cohort, 7,515 (approximately 75%) participated in the second follow-up examination, which included PWI-SF data and served as the baseline for the present analysis. Of these, 1,105 participants were excluded due to missing PWI-SF or LE8 data, a history of CVD at baseline, indeterminate CVD status during follow-up, or loss to follow-up (Figure 1). The final analytic sample included 6,410 participants, who were categorized into tertiles based on their PWI-SF scores (T1: lowest, T2: intermediate, T3: highest).

### 2.2. Study outcomes

CVD was defined as self-reported physician-diagnosed myocardial infarction, stroke, coronary artery disease, or congestive heart failure. Incident CVD cases were identified through biennial follow-up questionnaires and further validated through repeated in-depth personal interviews conducted by trained interviewers. A validation analysis within the KoGES cohort reported high agreement (93%) between self-reported diagnoses and medical record–based diagnoses, supporting the validity of this approach [17]. Participants with CVD at baseline were considered prevalent cases and excluded from the analysis. Incident CVD events were identified during follow-up when participants first reported a physician diagnosis of any of the specified CVD conditions.

Because the exact clinical diagnosis date was not available in the dataset, the index date was defined as the examination date of the survey wave in which the CVD diagnosis was first reported. Follow-up continued until the first reported CVD event or the last follow-up examination, whichever occurred first.

### 2.3. Measurement of psychological distress

Psychological distress was measured using the PWI-SF, an 18-item self-report questionnaire adapted from the General Health Questionnaire (GHQ) to screen for individuals at risk for poor mental health in Korean populations [18–21]. The PWI-SF assesses psychological distress by measuring both negative emotional responses to stress and the individual’s capacity to adapt and cope effectively. The scale comprises four subdomains identified through factor analysis: social performance and self-confidence (eight items), depression (three items), sleep disturbance and anxiety (three items), and general well-being and vitality (four items). Items are rated on a 4-point Likert scale (0 to 3), and the total score ranges from 0 to 54, with higher scores indicating greater psychological distress. The PWI-SF demonstrated good internal consistency in this study (Cronbach’s α = 0.88).

### 2.4. Assessment of LE8 CVH metrics and covariates

The LE8 score was calculated based on the AHA’s updated CVH metrics, which include four health behaviors (diet, physical activity, nicotine exposure, and sleep health) and four health factors (body mass index [BMI], blood lipids, blood glucose, and blood pressure) with modifications to account for population-specific characteristics and data availability, including the use of Korean-specific BMI criteria, adaptation of the diet component, and simplified scoring of nicotine exposure (Supplementary Table S1) [14]. In this study, BMI categories were defined according to the obesity classification of the Korean Society for the Study of Obesity, and corresponding LE8 scores were assigned based on these categories [22]. The diet component was assessed using the 16-item Modified Mediterranean Eating Pattern for Americans score [23]. Due to minimal consumption of olive oil among the Korean population, the olive oil item was excluded, resulting in a maximum possible score of 15 points. Nicotine exposure was assessed based on self-reported smoking status and secondhand smoke exposure. Participants who had never smoked were assigned 100 points, former smokers 50 points, and current smokers 0 points. Additionally, a 20-point deduction was applied to individuals exposed to secondhand smoke from household members. To determine the LE8 score, each component was assigned a point value ranging from 0 (worst) to 100 (best). The overall LE8 score was calculated as the composite sum of all eight component scores. Detailed definitions and scoring criteria for each LE8 component are provided in Supplementary Table S1. Covariates were selected a priori based on established literature on CVD risk factors and potential confounders [24]. Covariates included age, sex, residence area (urban, rural region), educational attainment (≤ elementary school, middle school, ≥ high school), and monthly household income (<**₩**1,500,000, ≥**₩**1,500,000).

### 2.5. Statistical analysis

Participants with missing data on key variables were excluded, and a complete case analysis was performed. Baseline characteristics across PWI-SF tertile groups were presented as means with standard deviations for continuous variables and as counts with percentages for categorical variables.

Kaplan–Meier survival curves were constructed to evaluate the cumulative incidence of CVD across PWI-SF tertiles, and comparisons were made using the log-rank test. Cox proportional hazards regression models were used to calculate hazard ratios (HRs) and 95% confidence intervals (CIs) for the association between PWI-SF scores and incident CVD, with adjustments for age, sex, residential area, educational level, household income, and LE8 CVH metrics. The lowest tertile (T1) of PWI-SF scores was used as the reference group. In addition, we performed a sensitivity analysis using previously suggested cut-offs for the PWI-SF (healthy ≤ 8, potential psychological distress 9–26, psychological distress group ≥ 27) [18]. The proportional hazards assumption was assessed using Schoenfeld residuals (*cox.zph* function in R), and no violation was observed (global test *p* > 0.05).

Subgroup analyses were conducted a priori based on established sociodemographic factors, stratified by age, sex, region, educational attainment, and household income. To investigate the independent contribution of psychological distress and individual CVH component to CVD risk, an additional multivariable Cox proportional hazards model was constructed. This model simultaneously included the eight individual metrics of the LE8 (diet, physical activity, nicotine exposure, sleep health, BMI, blood lipids, blood glucose, and blood pressure) and the PWI-SF score as a continuous variable, and socioeconomic factors (age, sex, residential region, educational attainment, and household income).

A *p*-value <0.05 was considered statistically significant. All statistical analyses were conducted using SAS Enterprise Guide version 9.4 (SAS Institute Inc., Cary, NC, USA) and R software version 3.5.3 (R Foundation for Statistical Computing, Vienna, Austria).

## 3. Results

### 3.1. Baseline characteristics of participants according to PWI-SF tertiles

A total of 6,410 participants were included in the analysis, with a mean age of 55.7 ± 8.7 years. Women comprised 52.7% of the study population, and 51.5% resided in Ansung, a rural area. The mean PWI-SF score was 17.8 ± 9.0 (range 0–54). Participants were categorized into tertiles based on their PWI-SF scores, ranging from the lowest (T1) to the highest (T3) (Table 1). Compared to those in the lowest tertile (T1), individuals in the highest tertile (T3) were more likely to be female and tended to have lower BMI, educational attainment, and household income. They also exhibited significantly lower overall LE8 CVH scores. Among the eight individual CVH components, reduced physical activity and poorer sleep health were associated with higher levels of psychological distress.

**Table 1.** Baseline characteristics of participants stratified by PWI-SF score.

Variables	Total (*n* = 6,410)	T1 (≤13) (*n* = 2,137)	T2 (14–21) (*n* = 2,137)	T3 (≥ 22) (*n* = 2,136)
PWI-SF (points)	17.8 ± 9.0	8.3 ± 3.8	17.2 ± 2.2	28.0 ± 5.6
Age (year)	55.7 ± 8.7	56.2 ± 8.7	55.1 ± 8.5	55.8 ± 8.8
Sex (n)				
Female	3,375 (52.7%)	919 (43.0%)	1,163 (54.4%)	1,293 (60.5%)
Male	3,035 (47.4%)	1,218 (57.0%)	974 (45.6%)	843 (39.5%)
BMI (kg/m^2^)	24.5 ± 3.1	24.7 ± 3.0	24.5 ± 3.0	24.4 ± 3.2
Residence area (n)				
Urban region (Ansan)	3,109 (48.5%)	1,031 (48.3%)	1,071 (50.1%)	1,007 (47.1%)
Rural region (Ansung)	3,301 (51.5%)	1,106 (51.8%)	1,066 (49.9%)	1,129 (52.9%)
Education (n)				
≤Elementary school	2,287 (35.7%)	691 (32.3%)	731 (34.2%)	865 (40.5%)
Middle school	1,306 (20.4%)	445 (20.8%)	416 (19.5%)	445 (20.8%)
≥High school	2,817 (44.0%)	1,001 (46.8%)	990 (46.3%)	826 (38.7%)
Monthly income (n)				
<₩1,500,000	2,964 (46.2%)	931 (43.6%)	937 (43.9%)	1,096 (51.3%)
≥₩1,500,000	3,446 (53.8%)	1,206 (56.4%)	1,200 (56.2%)	1,040 (48.7%)
LE8 CVH metric (points)	69.3 ± 11.8	69.5 ± 11.1	69.9 ± 11.9	68.5 ± 12.3
Diet	72.6 ± 13.9	72.7 ± 14.1	73.0 ± 13.6	72.0 ± 14.1
Physical activity	70.2 ± 45.8	74.8 ± 43.4	70.4 ± 45.7	65.3 ± 47.6
Nicotine exposure	68.1 ± 38.8	66.2 ± 38.9	69.8 ± 38.0	68.2 ± 39.5
Sleep health	79.6 ± 25.3	81.0 ± 24.1	79.9 ± 25.3	77.9 ± 26.3
BMI	62.2 ± 30.9	60.3 ± 30.6	62.4 ± 30.7	64.0 ± 31.1
Blood lipids	63.1 ± 28.3	63.7 ± 27.7	63.4 ± 28.5	62.1 ± 28.6
Blood glucose	81.9 ± 24.8	82.0 ± 24.1	82.4 ± 24.7	81.4 ± 25.6
Blood pressure	63.8 ± 31.5	62.4 ± 31.3	66.0 ± 31.4	63.2 ± 31.8

Participants were categorized into tertiles (T1–T3) according to their PWI-SF scores, with T1 representing the lowest tertile and T3 the highest. Values are shown as mean ± standard deviation or number (%). PWI-SF, Psychosocial Well-being Index Short Form; T, tertile; BMI, body mass index; LE8, Life’s Essential 8; CVH, cardiovascular health.

### 3.2. PWI-SF and CVD outcome

**Figure 2.**
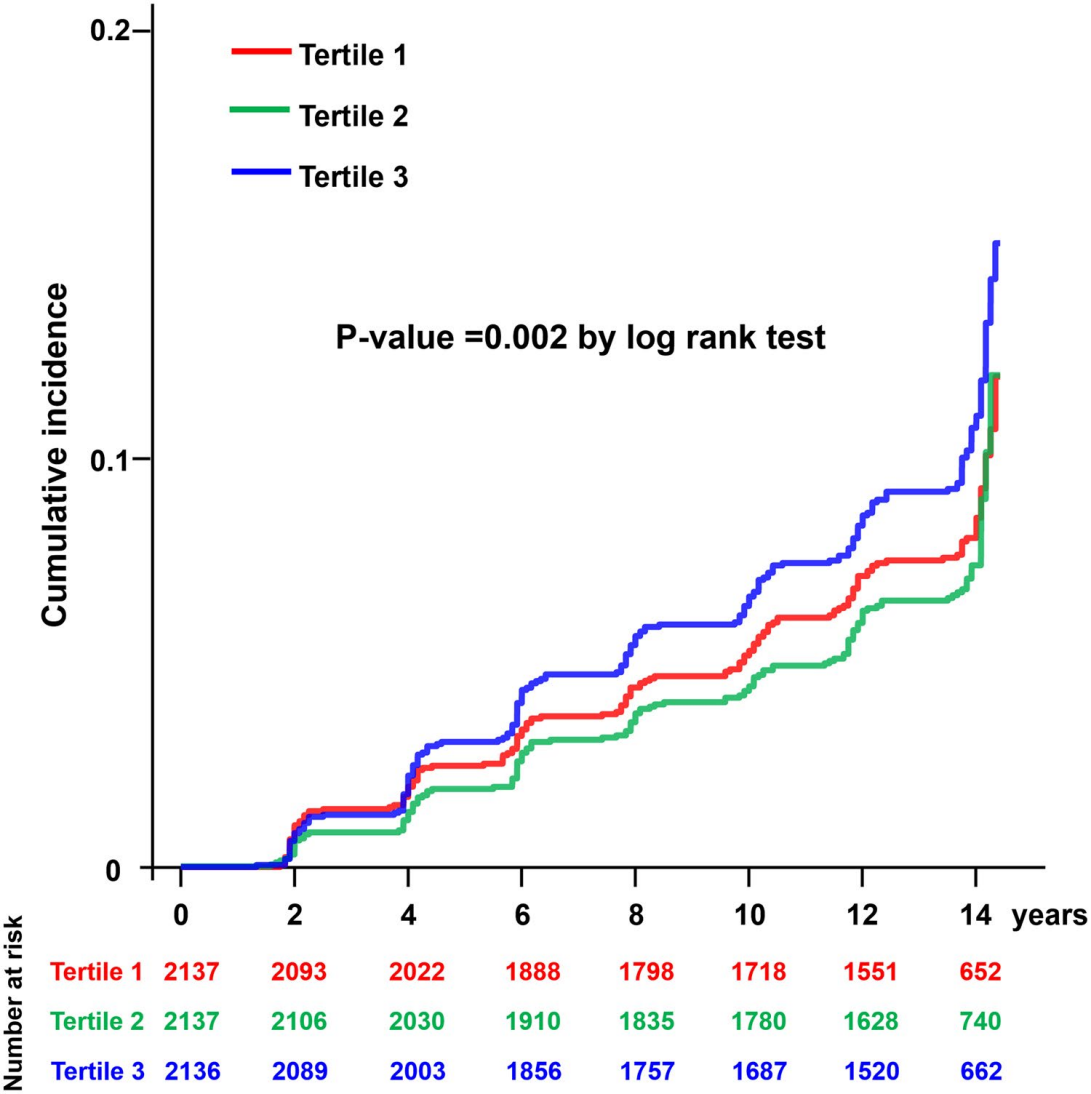
Kaplan–Meier curves for cumulative incidence of CVD by PWI-SF tertiles. Participants were categorized into three groups according to their PWI-SF scores: Tertile 1 (lowest psychological distress), Tertile 2 (intermediate psychological distress), and Tertile 3 (highest psychological distress). Over the follow-up period, individuals in the highest PWI-SF tertile exhibited a significantly higher cumulative incidence of CVD compared to those in the lowest tertile (log-rank *p* = 0.002). CVD, cardiovascular disease; PWI-SF, Psychosocial Well-being Index Short Form

During the median follow-up of 13.8 years (11.8–14.0 years), 500 cases of CVD occurred. Among these (including multiple events in some participants), stroke accounted for the largest proportion (49.8%), followed by coronary artery disease (37.8%), myocardial infarction (15.6%), and congestive heart failure (1.4%). The Kaplan–Meier curve analysis stratified by PWI-SF tertiles revealed that individuals in the highest PWI-SF tertile (T3) exhibited a significantly higher cumulative incidence of CVD compared to those in the lowest tertile (T1) (log-rank *p* = 0.002) (Figure 2).

In the Cox proportional hazards models, higher levels of psychological distress as assessed using the PWI-SF were significantly associated with an increased risk of CVD (Table 2). Compared with that of participants in the lowest tertile (T1) of PWI-SF scores, those in the highest tertile (T3) had a significantly higher risk of CVD in Model 1 (HR 1.387, 95% CI 1.121–1.717; *p* = 0.003), which was adjusted for age, sex, residential region, educational attainment, and household income. This association remained significant after further adjustment for LE8 CVH metrics (Model 2: HR 1.321, 95% CI 1.067–1.636; *p* = 0.011). The intermediate tertile (T2) did not show a significant increase in CVD risk compared with T1 in either Model 1 (HR 0.975, 95% CI 0.776–1.225; *p* = 0.828) or Model 2 (HR 0.963, 95% CI 0.767–1.210; *p* = 0.748).

**Table 2.** Association between PWI-SF score and risk of cardiovascular disease.

PWI-SF	Events at risk (n)	Model 1 HR (95% CI)	Adjusted *p*-value	Model 2 HR (95% CI)	Adjusted *p*-value
T1	156/2,137	1.000	Reference	1.000	Reference
T2	144/2,137	0.975 (0.776–1.225)	0.828	0.963 (0.767–1.210)	0.748
T3	200/2,136	1.387 (1.121–1.717)	0.003	1.321 (1.067–1.636)	0.011

T1, lowest tertile (reference group); T2, intermediate tertile; T3, highest tertile. Analyses for Model 1 were adjusted for potential confounders, including age, sex, residential region, educational attainment, and household income. Analyses for Model 2 were adjusted for the covariates in Model 1 as well as the Life’s Essential 8 cardiovascular health metrics. The proportional hazards assumption was satisfied (Global *p* = 0.179 for Model 1 and 0.208 for Model 2). PWI-SF, Psychosocial Well-being Index Short Form; HR, hazard ratio; CI, confidence interval; T, tertile.

In a sensitivity analysis using previously suggested cut-offs for the PWI-SF, compared with the healthy group (PWI-SF score ≤8), the distress group (PWI-SF score ≥ 27) was associated with an increased risk of CVD (Model 2: HR 1.431, 95% CI 1.048–1.954; *p* = 0.024) (Supplementary Table S2). The results were consistent with the main findings.

**Figure 3.**
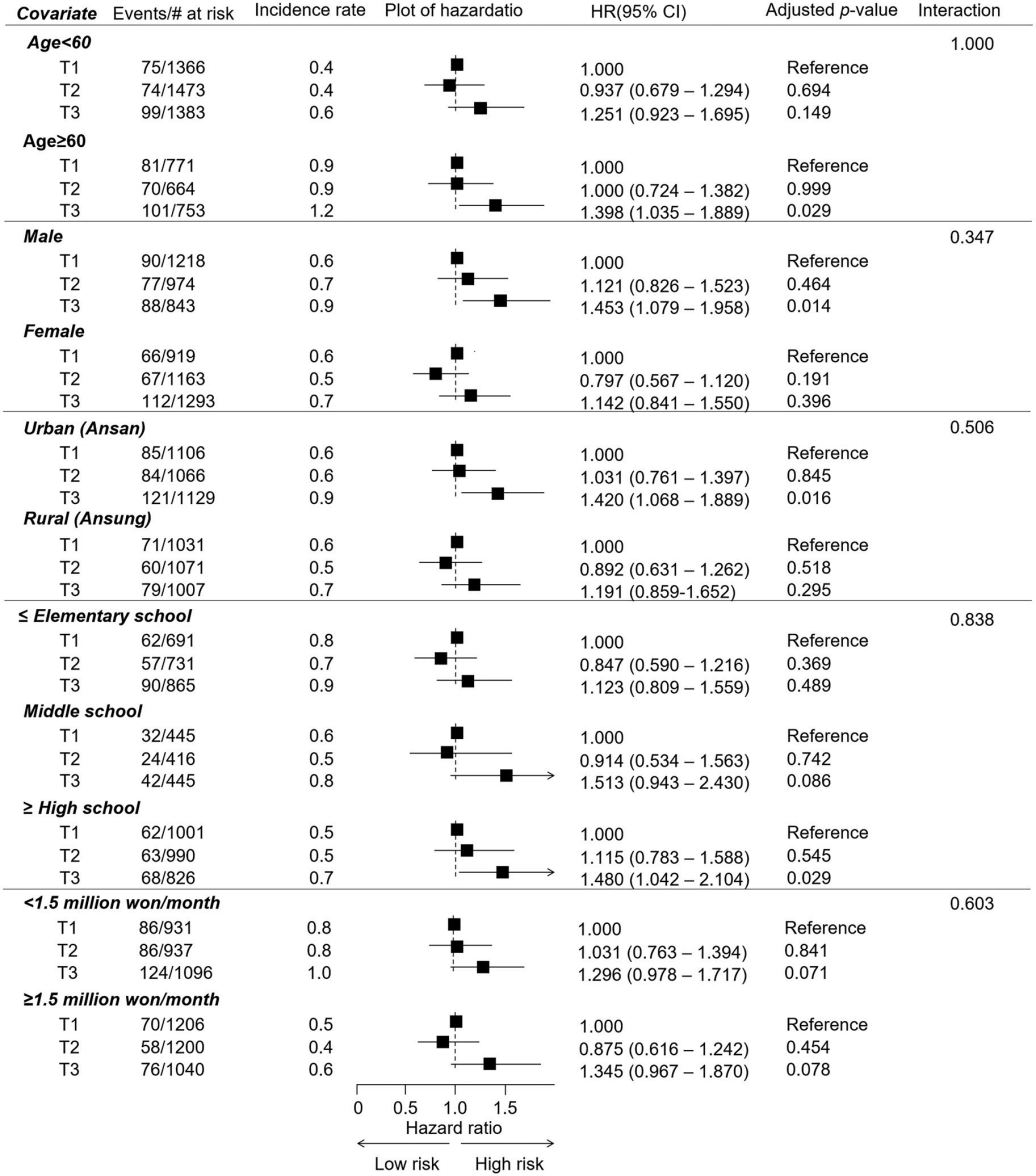
Subgroup analyses of the association between the PWI-SF and risk of incident CVD. HRs and 95% CIs for each PWI-SF tertile group were estimated using Cox proportional hazards models. Analyses were stratified by age, sex, residence area, educational attainment, and household income, and were mutually adjusted for these covariates as well as the LE8 CVH metrics. PWI-SF, Psychosocial Well-being Index Short Form; CVD, cardiovascular disease; HRs, hazard ratios; CIs, confidence intervals; LE8, Life’s Essential 8; CVH, cardiovascular health; T, tertile of PWI-SF scores (T1, lowest [reference group]; T2, intermediate; T3, highest)

Subgroup analyses indicated that the association between PWI-SF scores and CVD outcomes remained generally consistent across strata of age, sex, residential area, educational attainment, and household income. No significant interactions were observed, suggesting the absence of effect modification across these subgroups (all *p* for interaction > 0.05; Figure 3).

**Figure 4.**
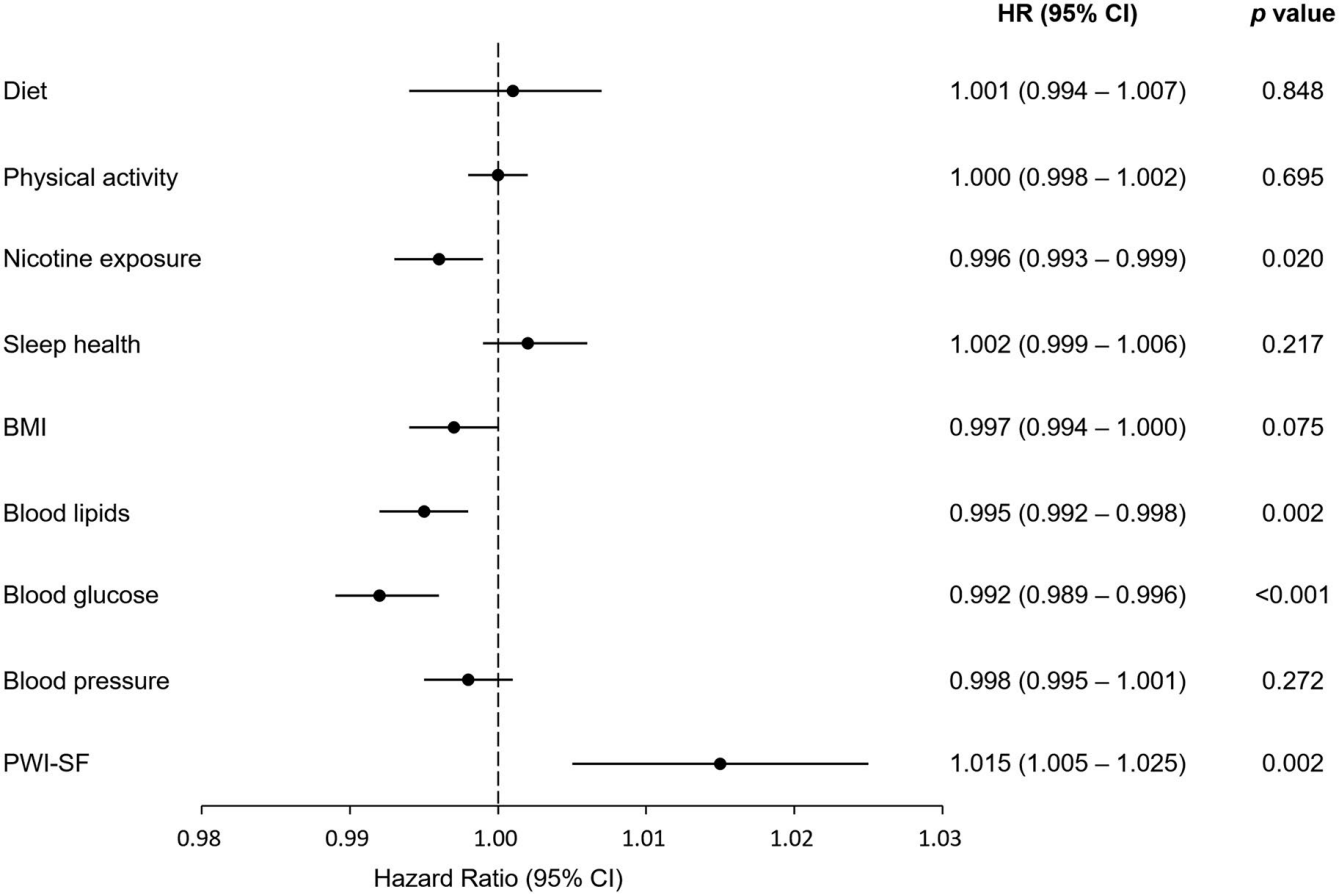
Forest plot showing associations between LE8 CVH metrics, PWI-SF score, and CVD incidence. Individual components of LE8 CVH metrics and PWI-SF were adjusted for potential confounders, including sex, age, residence area, educational attainment, and household income. CVD, cardiovascular disease; PWI-SF, Psychosocial Well-being Index Short Form; HR, hazard ratio; CI, confidence interval; BMI, body mass index; LE8, Life’s Essential 8; CVH, cardiovascular health

We further examined whether the association between psychological distress and CVD incidence remained robust when individual LE8 components were considered simultaneously. In this comprehensive model (Figure 4), which included all eight individual LE8 metrics and PWI-SF scores as a continuous variable, higher PWI-SF scores remained significantly associated with an increased risk of CVD (HR 1.015, 95% CI 1.005–1.025; *p* = 0.002). Among the individual LE8 components, healthy levels of blood glucose (HR 0.992, 95% CI 0.989–0.996; *p* < 0.001), healthy levels of blood lipids (HR 0.995, 95% CI 0.992–0.998; *p* = 0.002), and avoidance of nicotine exposure (HR 0.996, 95% CI 0.993–0.999; *p* = 0.020) were identified as significant independent protective factors against CVD incidence. Notably, the independent association between psychological distress and CVD risk persisted even after comprehensive adjustment for each individual LE8 metric.

## 4. Discussion

In this large community-based prospective cohort study, higher levels of psychological distress, assessed using the PWI-SF, were associated with an increased risk of incident CVD over 14 years of follow-up. This association remained significant after adjustment for sociodemographic characteristics and LE8 CVH metrics. These findings suggest that psychological distress may capture unique dimensions of cardiovascular risk not fully reflected by existing CVH metrics. From a clinical and public health perspective, the assessment of psychological distress may therefore enhance cardiovascular risk stratification by identifying individuals who may benefit from more comprehensive cardiovascular risk management.

Our findings are consistent with previous large-scale prospective studies that have identified psychological distress as a significant risk factor for adverse cardiovascular outcomes. The Whitehall II study [10,25], which used the GHQ—a multidimensional instrument conceptually similar to the PWI-SF used in our study—reported that psychological distress was associated with an increased risk of coronary heart disease. Although the Whitehall II study provided important early evidence linking psychological distress to cardiovascular outcomes, its findings were derived from an occupational cohort of civil servants, which may limit their generalizability to the broader population. In the present study, we extend this evidence by examining this association in a large community-based cohort drawn from the general population. Similarly, the 45 and Up Study [12], a large Australian cohort including more than 200,000 adults, used the Kessler Psychological Distress Scale (K10) and demonstrated a dose–response relationship between the severity of psychological distress and the incidence of myocardial infarction and stroke. Hamer et al. reported that psychological distress measured using the GHQ-12 was associated with an increased risk of cardiovascular events in age- and sex-adjusted models in the Scottish Health Survey [26]. However, the association was no longer statistically significant after adjustment for behavioral risk factors, suggesting that behavioral factors may partly mediate the relationship between psychological distress and cardiovascular risk. In contrast, in the present study the association between psychological distress and incident CVD remained significant even after adjustment for the LE8 CVH metrics and sociodemographic covariates. These findings suggest that psychological distress may represent an additional dimension of cardiovascular risk that is not fully captured by current LE8 CVH metrics.

Gaffey et al. recently proposed expanding the LE8 CVH metrics to include mental health, introducing ‘Life’s Crucial 9,’ with psychological health as the ninth essential component of CVH metrics [27]. Our findings provide empirical support for this evolving perspective. Although the present study specifically examined psychological distress, our results suggest that psychological health may warrant consideration as an independent domain of CVH rather than merely a contextual modifier in future CVH assessments.

Psychological distress may influence CVD through multifactorial pathways involving complex interactions between the brain, endocrine system, autonomic nervous system, and immune system [7,28,29]. Chronic stress disrupts adaptive stress-response pathways, particularly through repeated activation of corticolimbic brain regions such as the amygdala, hippocampus, and medial prefrontal cortex [30]. These regions regulate the hypothalamic–pituitary–adrenal axis and the autonomic nervous system, which coordinate neuroendocrine and sympathetic responses to stress [29]. Sustained stimulation of these systems leads to elevated circulating levels of cortisol and catecholamines, which increase blood pressure and heart rate, activate prothrombotic pathways, and contribute to endothelial dysfunction [7]. Additionally, chronic stress promotes systemic inflammation by upregulating cytokines and adhesion molecules, thereby accelerating atherosclerotic processes and contributing to vascular injury and plaque instability [7,29]. These physiological effects provide a plausible biological basis for the observed association between elevated psychological distress and increased CVD risk, independent of conventional behavioral and metabolic risk factors.

Brief self-report questionnaires provide a practical approach to assessing psychological distress in large epidemiological studies, particularly in clinical and community settings where time and resources are limited. In this study, we utilized the PWI-SF, which has been widely validated and established as a reliable instrument for assessing psychological distress in Korean populations. The PWI-SF has been applied across diverse settings, including college students, stroke survivors, automobile industry workers, and firefighters [21,31–33]. Our findings are consistent with previous studies suggesting that brief self-report psychological distress screening instruments are associated with cardiovascular outcomes [34].

From a public health perspective, our findings suggest the potential importance of integrating psychological distress assessments into routine cardiovascular risk evaluation, particularly in community settings. Traditional risk models and behavioral metrics, such as LE8 CVH metrics, may not fully capture the adverse effects of psychological distress, and overlooking this dimension may lead to an incomplete assessment of cardiovascular risk in vulnerable populations. Further research is needed to determine how assessment of psychological distress can be incorporated into cardiovascular risk assessment and prevention strategies.

The present study has several strengths. First, to the best of our knowledge, this is the first large-scale, community-based prospective study to demonstrate the association between psychological distress and incident CVD, independent of LE8 CVH metrics. Second, the use of a well-characterized longitudinal cohort with a long follow-up period and comprehensive covariate data enhances the validity and generalizability of our findings. Third, the prospective design allowed for the assessment of psychological distress prior to the onset of CVD, enabling a clear temporal sequence that supports causal inference.

However, several limitations of this study should be acknowledged. First, psychological distress was assessed using the PWI-SF, a validated and widely used self-report instrument in Korean populations. Although this approach is commonly used in epidemiological studies, the use of self-reported measures may introduce reporting bias or misclassification. Second, CVD events were identified based on participants’ self-reports of physician-diagnosed conditions, which may also be subject to recall bias or misclassification. Third, although we adjusted for a broad range of sociodemographic and clinical factors, residual confounding cannot be entirely excluded. In addition, the use of complete case analysis due to missing data may have introduced potential selection bias. Finally, our findings may not be generalizable beyond the Korean adult population, and further studies in other ethnic and cultural contexts are warranted.

In conclusion, higher levels of psychological distress were associated with an increased risk of CVD in a large Korean community-based cohort, even after adjusting for LE8 CVH metrics and sociodemographic factors. These findings suggest that psychological distress may represent an additional dimension of cardiovascular risk beyond conventional behavioral and biological determinants. Incorporating brief, validated assessments of psychological distress into cardiovascular risk evaluation may help improve risk assessment in community settings. Future studies are needed to evaluate how psychological distress assessment can be integrated into clinical and public health prevention strategies.

## Supplementary Material

Supplementary material 1_Revision.docx

Supplementary material 2_STROBE CHECKLIST.docx

## Data Availability

The data that support the findings of this study are available from the Korean Genome and Epidemiology Study (KoGES). However, restrictions apply to the availability of these data, which were used under license for the current study, and are not publicly available. Data are available from the authors upon reasonable request and with permission of the Data Access Committee in the Clinical & Omics Data Archive (CODA) of the Korea Disease Control and Prevention Agency.
