# Supplementary material for: Psychological distress and incident cardiovascular disease independent of life’s essential 8: a prospective cohort study
Source: Ann Med. 2026 May 4;58(1):2665515. doi: 10.1080/07853890.2026.2665515 (PMC13142179; doi:10.1080/07853890.2026.2665515)
Supplement: Supplementary material 1_Revision.docx [file IANN_A_2665515_SM5079.docx]

**Supplementary Table S1. Measurement criteria and quantitative assessment of Life’s Essential 8 cardiovascular health metrics.**

| **LE8 metrics** | **Measurements** | **Levels** | **Quantitative points** |
| --- | --- | --- | --- |
| **Diet (points)** | Self-reported daily intake of a DASH-style eating pattern, as assessed by the MEPA score^1)^ | 14–15 | 100 |
|  |  | 11–13 | 80 |
|  |  | 7–10 | 50 |
|  |  | 3–6 | 25 |
|  |  | 0–2 | 0 |
| **Physical activity (min)** | Self-reported weekly minutes of moderate or vigorous physical activity | ≥150 | 100 |
|  |  | 120–149 | 90 |
|  |  | 90–119 | 80 |
|  |  | 60–89 | 60 |
|  |  | 30–59 | 40 |
|  |  | 1–29 | 20 |
|  |  | 0 | 0 |
| **Nicotine exposure** | Self-reported use of cigarette at any age, or exposure to secondhand smoke. A deduction of 20 points (unless score is 0) was applied for individuals living with an active indoor smoker at home | Non smoker | 100 |
|  |  | Former smoker | 50 |
|  |  | Current smoker | 0 |
| **Sleep health (h)** | Self-reported average hours of sleep per night | 7–<9 | 100 |
|  |  | 9–<10 | 90 |
|  |  | 6–<7 | 70 |
|  |  | 5–<6 or ≥10 | 40 |
|  |  | 4–<5 | 20 |
|  |  | <4 | 0 |
| **BMI (Kg/m^2^)** | BMI (kg/m^2^) was calculated using measured weight (kg) and height (m)^(2)^ | <23 | 100 |
|  |  | 23.0–24.9 | 70 |
|  |  | 25.0–29.9 | 30 |
|  |  | 30.0–34.9 | 15 |
|  |  | ≥35 | 0 |
| **Blood lipids (mg/dL)** | Non-HDL cholesterol was calculated using measured plasma total cholesterol-HDL cholesterol levels. Subtract 20 points if treated level | <130 | 100 |
|  |  | 130–159 | 60 |
|  |  | 160–189 | 40 |
|  |  | 190–219 | 20 |
|  |  | ≥220 | 0 |
| **Blood glucose** | FBG or HbA1c levels were measured. | No history of diabetes and FBG <100 mg/dL (or HbA1c <5.7%) | 100 |
|  |  | No diabetes and FBG 100–125 mg/dL (or HbA1c 5.7–6.4%) | 60 |
|  |  | Diabetes with HbA1c <7.0% | 40 |
|  |  | Diabetes with HbA1c 7.0–7.9% | 30 |
|  |  | Diabetes with HbA1c 8.0–8.9% | 20 |
|  |  | Diabetes with HbA1c 9.0–9.9% | 10 |
|  |  | Diabetes with HbA1c ≥10.0% | 0 |
| **Blood pressure (mmHg)** | Systolic and diastolic BPs were appropriately measured.  Subtract 20 points if treated level | <120/<80 | 100 |
|  |  | 120–129/<80 | 75 |
|  |  | 130–139/80–89 | 50 |
|  |  | 140–159/90–99 | 25 |
|  |  | ≥160/≥100 | 0 |

^1)^ Olive oil item was excluded from the evaluation due to its low consumption among the Korean population, resulting in a maximum possible score of 15 points.

^2)^ BMI criteria for assigning LE8 quantitative points were modified according to the obesity classification defined by the Korean Society for the Study of Obesity.

LE8, Life’s Essential 8; DASH, Dietary Approaches to Stop Hypertension; MEPA, Mediterranean Eating Pattern for Americans; BMI, body mass index; HDL, high-density lipoprotein; FBG, fasting blood glucose; HbA1c, hemoglobin A1c; BPs, blood pressures.

**Supplementary Table S2. Supplementary Table S3. Sensitivity Analysis of the Association Between Cut-off–Based Categories of PWI-SF and Cardiovascular Disease**

| PWI-SF | Events at risk (n) | Model 1  HR (95% CI) | Adjusted *p*-value | Model 2  HR (95% CI) | Adjusted *p*-value |
| --- | --- | --- | --- | --- | --- |
| Healthy | 67/940 | 1.000 | Reference | 1.000 | Reference |
| Potential distress | 328/4,386 | 1.170 (0.898–1.524) | 0.245 | 1.150 (0.883–1.499) | 0.300 |
| Distress | 105/1,084 | 1.520 (1.114–2.075) | 0.008 | 1.431 (1.048–1.954) | 0.024 |

PWI-SF categories were defined using previously suggested clinical cut-offs: healthy (≤ 8), potential psychological distress (9–26), and psychological distress (≥27). Analyses for Model 1 were adjusted for potential confounders, including age, sex, residential region, educational attainment, and household income. Analyses for Model 2 were adjusted for the covariates in Model 1 as well as the Life’s Essential 8 cardiovascular health metrics. PWI-SF, Psychosocial Well-being Index Short Form; HR, hazard ratio; CI, confidence interval
